# Normal Thermostability of p.Ser113Leu and p.Arg631Cys Variants of Mitochondrial Carnitine Palmitoyltransferase II (CPT II) in Human Muscle Homogenate

**DOI:** 10.3390/metabo12111141

**Published:** 2022-11-19

**Authors:** Pushpa Raj Joshi, Maria Gräfin zu Stolberg-Stolberg, Leila Motlagh Scholle, Beate Meinhardt, Elena Pegoraro, Stephan Zierz

**Affiliations:** 1 Department of Neurology, Martin-Luther-University Halle-Wittenberg, 06120 Halle (Saale), Germany; 2 Institute of General Practice and Family Medicine, Martin-Luther-University Halle-Wittenberg, 06112 Halle (Saale), Germany; 3 Genowans Diagnostic, 06120 Halle (Saale), Germany; 4 Department of Neurosciences, University of Padova, 35121 Padova, Italy

**Keywords:** CPT II, muscle, mitochondria, thermolability, malonyl-CoA, cardiolipin

## Abstract

Previous fibroblast and recombinant enzyme studies showed a markedly thermolabile p.Ser113Leu variant compared to the wild-type (WT) in muscle carnitine palmitoyltransferase II (CPT II) deficiency. Additionally, it has been shown that cardiolipin (CLP) stimulated or inhibited the p.Ser113Leu recombinant variant depending on the pre-incubation temperatures. In this study, the thermolabilities of mitochondrial enzyme CPT II in muscle homogenates of patients with the p.Ser113Leu (n = 3) and p.Arg631Cys (n = 2) variants were identified to be similar to that of WT. Pre-incubation with CLP on ice stimulated the WT enzyme more than both variants. However, CLP stimulated the variants and WT at 46 °C to about 6–18-fold. The present data indicate that the thermostability of CPT II variant in muscle homogenate is similar to that of WT. This is in contrast to the increased thermolability of enzymes derived from fibroblast and that of recombinant enzymes. Hence, it can be speculated that the disruption of the compartmentation in muscle homogenate mediates a protective effect on the thermolability of the native variant. However, the exact mechanism remains unclear. However, the activating effect of CLP on CPT II in muscle homogenate seems to align with those on recombinant enzymes.

## 1. Introduction

Transporting long-chain fatty acids into the mitochondria requires, besides other enzymes, carnitine palmitoyltransferase II (CPT II) located at the inner mitochondrial membrane [1,2]. CPT II deficiency presents with three phenotypes: lethal neonatal form [3,4,5,6], severe infantile hepatocardiomuscular form [3,7,8,9] and the most frequent classical myopathic form with a mild phenotype. The myopathic form is clinically characterized by recurrent episodes of myalgia, muscle weakness and rhabdomyolysis triggered by prolonged exercise, fasting, exposure to cold, fever and emotional stress. Persistent muscle weakness is rarely seen in CPT II deficiency [10,11,12,13]. Neurological examination and muscle biopsy are usually normal between the attacks [10,11]. A common p.Ser113Leu mutation in the CPT2 gene is identified in about 90% of patients in at least one allele as well as about 100 different rare mutations are also reported in the CPT2 gene [10,14].

Different studies have attempted to explain the ambiguity regarding the clinical and biochemical consequences of mutant CPT II enzymes. An abnormally regulated enzyme rather than a reduced catalytic activity in CPT II deficient patients has been repeatedly demonstrated [14,15,16,17]. Olpin et al. have found a reduced palmitoyl oxidation rate at 41 °C compared to 37 °C in fibroblasts of CPT II deficient patients. Markedly reduced thermostability of the enzyme was also confirmed in recombinant enzymes in the p.Ser113Leu variant [17,18]. This thermolability reduced the enzyme activity of the p.Ser113Leu recombinant variant after 15 min at 40 °C to about 5% of the initial activity in contrast to almost retained enzyme activity in the wild-type. At 45 °C for 15 min, the enzyme activity of the wild-type was reduced by about 20% whereas there was a complete loss of enzyme activity of the recombinant p.Ser113Leu variant [19]. Therefore, it has been speculated that most of the trigger factors of CPT II deficiency are related to increased body temperature. Previously, the protein stabilizing diphosphatidylglycerol cardiolipin (CLP) has been shown to stabilize the enzyme activity of recombinant CPT II [20,21]. 

In the present study, the thermostabilities of the CPT II variants with the common p.Ser113Leu mutation and the rare p.Arg631Cys variant were evaluated in muscle homogenates. In addition, the effect of cardiolipin (CLP) on the enzyme activities at 0 °C, 40 °C and 46 °C was analysed. 

## 2. Patients and Controls

The study was performed on muscle homogenates of five patients with the muscle form of CPT II deficiency. Three patients were homozygous for the common p.Ser113Leu mutation (two males and one female), and two patients had a rare p.Arg631Cys mutation in a homozygous state (both males). The mean age of patients at the time of muscle biopsy was 28.8 ± 5.7 years (range: 19–44 years). Detailed epidemiological and molecular data of patients are listed in Table 1. The data were compared to 13 controls (6 males, 7 females; mean age: 49.07 ± 14.08 years). Controls were biopsied for the suspected neuromuscular disease but ultimately found no neuromuscular disease, including the CPT II deficiency. 

Muscle homogenates of two to five patients and three to seven controls were used as per availability in each set of experiments. The exact number of patients and controls are annotated in the legends of individual figures. 

Written informed consent for analyzing muscle biopsy samples was obtained from all patients and controls. All experiments were carried out in accordance with The Code of Ethics of the World Medical Association (Helsinki Declaration).

## 3. Materials and Methods

### 3.1. CPT Assay

For biochemical examination, the frozen muscle was homogenised by dilution with Chappel–Perry-medium (1:30) containing KCl (100 mM), Tris (50 mM), MgCl_2_ (5 mM) and EDTA (1 mM) using a glass-homogeniser (Thermo Fischer Scientific, MA, USA) on ice. Pre-incubation was done on ice at 0 °C, and higher temperature incubation up to 46 °C was done using a thermomixer (Eppendorf SE, Hamburg, Germany).

Thermolabilities of mutant (p.Ser113Leu and p.Arg631Cys) and wild-type CPT II were evaluated:

(i) For 6 min at different temperatures (30 °C, 37 °C, 40 °C, 45 °C, 49 °C);
(ii) At 40 °C and 46 °C for up to 15 min (0, 5, 7, 10, 15 min).

CPT activity was measured using the previously established isotope forward assay [22]. 

In the experiments with cardiolipin (CLP), the homogenate was pre-incubated with CLP (final concentration 0.25 mM) at different temperatures.

### 3.2. Protein Determination

Non-collagenous protein content was measured using the conventional bicinchoninic acid (BCA) assay developed by Smith et al. [23].

## 4. Results

### 4.1. Thermolability at Different Temperatures and Times

**Figure 1:**
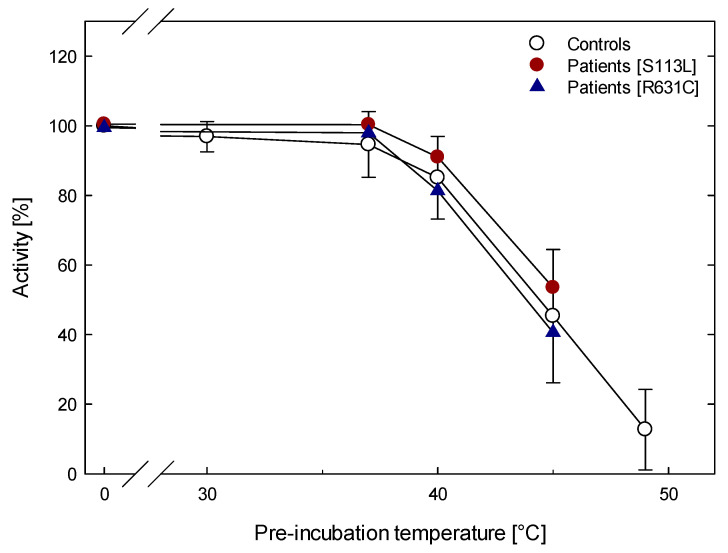
CPT activity in muscle homogenates of wild-type (n = 7) and mutant (n = 5) [p.Ser113Leu (n = 3) and p.Arg631Cys (n = 2)] with pre-incubating at increasing temperatures for 6 min. The error bars correspond only to data of wild-type.

The wild-type and mutant enzyme activities after pre-incubation for six minutes at 0 to 37 °C were almost stable. The activities of controls and patients (p.Ser113leu and p.Arg631Cys) decreased by 10–20% at 40 °C and dropped to less than 50% at 46 °C. The 50% loss of enzyme activity (T50) was perceived between 43 to 45 °C for controls and both p.Arg631Cys and p.Ser113Leu variants (Figure 1).

Activities of mutant and wild-type enzymes progressively decreased after incubating at 40 °C. At 40 °C for 15 min, the activities of both mutants decreased by about 15%, and the activity of controls decreased by 20%. At 46 °C for 15 min, the mutant and wild-type enzyme activities drastically decreased to about 10% of the initial enzyme activity at 0 °C.

**Figure 2:**
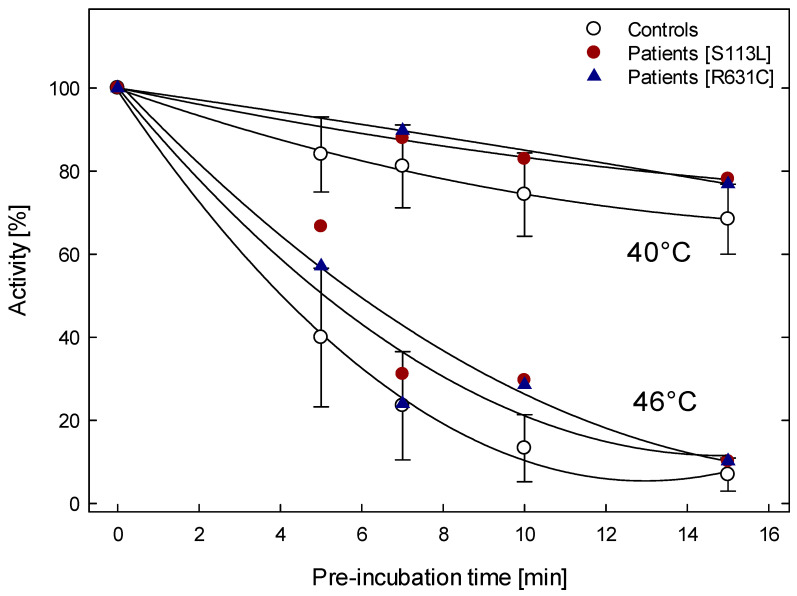
CPT activity in muscle homogenates of wild-type and patients with pre-incubating at 40 °C [wild-type (n = 5) and mutant (n = 4); p.Ser113Leu (n = 3) and p.Arg631Cys (n = 1)] and 46 °C [wild-type (n = 8) and mutant (n = 4); p.Ser113Leu (n = 2) and p.Arg631Cys (n = 2)] for up to 15 min. The error bars correspond only to data of the wild-type.

To summarize, at both temperatures (40 °C and 46 °C) and various incubation times, the activities of patients tended to be slightly but not significantly more stable than the controls (Figure 2).

### 4.2. Preincubation with Cardiolipin (CLP)

Pre-incubation with CLP on ice stimulated the wild-type enzyme by almost 40% in comparison to only 10% stimulation of both mutated enzymes (*p* < 0.001). At 40 °C with CLP, the CPT activity in patients was reduced to about 50% of the activities with CLP at 0°C but only to 84% in the wild-type (*p* = 0.025). However, at 46 °C, CLP stimulated the enzyme activities of both the mutants and wild-type up to 6–18-fold (Table 2).

## 5. Discussion

Patients with CPT II deficiency do not have persistent muscle weakness and no significant intramuscular lipid accumulation. Instead, they suffer from intermittent attacks of muscle weakness, myalgia and rhabdomyolysis triggered by prolonged exercise, fasting, exposure to cold, fever and emotional stress [10,11]. Most of these trigger factors are associated with increased body temperature. This thermogenesis is caused mainly due to fatty acid metabolism in skeletal muscle [24,25,26]. Therefore, it was intriguing to speculate that the increased thermolability of the recombinant purified human CPT II variant might explain why these trigger factors cause the attacks [19]. Previously, a marked thermal instability in the rate of palmitate oxidation has been shown in fibroblasts of patients with muscle form of CPT II deficiency at 41 °C compared to 37 °C [18].

Moreover, recombinant enzyme studies have revealed extreme thermolability of the p.Ser113Leu variant compared to the wild-type [17,19]. It has been shown that at 40 °C for 15 min, the enzyme activity of the p.Ser113Leu recombinant variant was reduced to about 5% of the initial activity in contrast to almost retained enzyme activity in wild-type. In fact, at 40 °C, the time for 50% enzyme activity (t1/2) was 4 min. The activity of the wild-type was very stable at 40 °C; the t1/2 was not reached even after 4 h [19]. 

In contrast to recombinant enzyme studies, present results showed unaltered thermal stability of the total CPT activity (CPT I and II) in muscle homogenates of patients with the p.Ser113Leu and p.Arg631Cys variants compared to normal controls. It can be speculated that the normal thermostability of the mutant enzyme in muscle homogenates might be an artefact upon disruption of the mitochondrial compartment, possibly mediated by the mitochondrial matrix compartment or the cytosolic compartment. Since it has been shown that acyl-L-carnitines with more than 10 carbons in the acyl side-chain mitigates the thermoinstability of the p.Ser113Leu variant [19], it cannot be excluded that these metabolites or other, so far, not identified substances can obtain access to CPT II in homogenized muscle. Therefore, it is still unclear whether the thermolability of the CPT II enzyme in vivo is similar to that in muscle homogenates or is similar to that in the recombinant enzyme or fibroblast-derived enzyme. 

The phospholipid environment of the mitochondrial inner membrane contains large amounts of CLP. Previously, CLP has been shown to activate recombinant non-purified rat CPT II enzyme almost fourfold at a physiological temperature [20]. Two other studies showed the activating ability of CLP for recombinant purified human CPT II in vitro, although the effects of CLP on both wild-type and variants were rather inconsistent [21,27]. Meinhard, et al. showed a stabilizing effect of CLP on the enzyme activities of the wild-type and the variant in vitro at 30 °C and 42 °C [21]. In contrast, Motlagh Scholle et al. found enzyme-activating effects only at 37 °C on wild-type, but the p.Ser113Leu variant was almost abolished by CLP [27]. This discrepancy can be explained by applying previous methodological approaches to the enzyme measurements. Meinhardt et al. replaced the micelle-forming detergent, β-d-glucopyranoside (β-OG), with an assay buffer before enzyme measurement [21]. This buffer replacement was not performed in the study by Motlagh Scholle et al. [27]. The stabilization of recombinant purified human CPT II enzymes by β-OG was also shown in the former study [21]; thus, it could be speculated that β-OG masked the stabilizing effect of CLP in the latter study [27].

In the present study on muscle homogenate, CLP stimulated both wild-type and mutant enzymes (p.Ser113Leu and p.Arg631Cys) at 46 °C but not at 40 °C. At 46 °C, CLP stimulated both the variants and wild-type to about 6–18-fold, similar to the results obtained with recombinant non-purified rat CPT II [20]. It can be speculated that the conformational changes of the enzyme and the membrane at higher temperatures might facilitate the binding of CLP. Moreover, a hydrophobic site of CPT supports the anchoring of the enzyme in the mitochondrial membrane responsible for binding the phospholipids and palmitoyl-CoA together [28]. Further studies with incubation of the recombinant enzyme with various cellular subtractions would help provide insight into the exact mechanism that might increase the thermostability of the variant.

In conclusion, the present study shows that the mutant CPT II variants in muscle homogenate exhibit the same thermostability as the wild-type variant in contrast to the increased thermolability of enzymes derived from fibroblasts and recombinant-produced enzymes. Moreover, the activating effect of CLP on CPT II in muscle homogenate seems to be similar to those on recombinant enzymes.

## Figures and Tables

**Table 1:** Epidemiological and molecular genetic data of patients.

Patients (m/f)	Age at Biopsy (Years)	Genotype
1 (f)	19	p.Ser113Leu/p.Ser113Leu
2 (m)	21	p.Ser113Leu/p.Ser113Leu
3 (m)	36	p.Ser113Leu/p.Ser113Leu
4 (m)	24	p.Arg631Cys/p.Arg631Cys
5 (m)	44	p.Arg631Cys/p.Arg631Cys

m: Male, f: Female.

**Table 2:** Effect of cardiolipin (CLP) (0.25 mM) on CPT II activities in muscle homogenates of controls and patients with the p.Ser113Leu and p.Arg631Cys variants. Values are expressed as the ratio of the activity with over without pre-incubation with CLP for 7 and 15 min at 40 °C and 46 °C. The number of patients are given in parentheses. The data of controls are shown as mean (±SD) and the data of patients are shown as means wherever applicable.

Pre-Incubation Temperature [°C]	Controls	Patients	
		p.Ser113Leu	p.Arg631Cys
0 °C	1.39 ± 0.13 (n = 5)	1.14 (n = 3)	1.12 (n = 2)
40 °C			
7 min	1.16 ± 0.07 (n = 3)	0.64 (n = 2)	0.61 (n = 1)
15 min	0.94 ± 0.06 (n = 3)	0.57 (n = 2)	0.59 (n = 1)
46 °C			
7 min	4.79 ± 1.66 (n = 3)	3.92 (n = 1)	5.75 (n = 1)
15 min	9.22 ± 4.37 (n = 3)	20.47 (n = 1)	9.02 (n = 1)

## Data Availability

The data presented in this study are available on reasonable request from the corresponding author. The data are not publicly available due to confidentiality of patients and controls.
